# High Psychosocial Work Demands, Decreased Well-Being, and Perceived Well-Being Needs Within Veterinary Academia During the COVID-19 Pandemic

**DOI:** 10.3389/fvets.2021.746716

**Published:** 2021-10-18

**Authors:** Hayley McKee, Basem Gohar, Ryan Appleby, Behdin Nowrouzi-Kia, Briana N. M. Hagen, Andria Jones-Bitton

**Affiliations:** ^1^Department of Population Medicine, The University of Guelph, Guelph, ON, Canada; ^2^Department of Psychology, The University of Guelph, Guelph, ON, Canada; ^3^Centre for Research in Occupational Safety and Health, Laurentian University, Sudbury, ON, Canada; ^4^Department of Clinical Studies, The University of Guelph, Guelph, ON, Canada; ^5^Department of Occupational Science and Occupational Therapy, Temerty Faculty of Medicine, The University of Toronto, Toronto, ON, Canada

**Keywords:** psychosocial work demands, veterinary well-being, COVID-19, academia well-being, mental health, stress

## Abstract

Higher psychosocial work demands in veterinary and academic professions are associated with decreased occupational, physical, and mental well-being. COVID-19 introduced far-reaching challenges that may have increased the psychosocial work demands for these populations, thereby impacting individual- and institutional-level well-being. Our objective was to investigate the psychosocial work demands, health and well-being, and perceived needs of faculty, staff, residents and interns at the Ontario Veterinary College, in Ontario, Canada, during COVID-19. A total of 157 respondents completed a questionnaire between November 2020 and January 2021, that included the Third Version of the Copenhagen Psychosocial Questionnaire (COPSOQ-III) and open-text questions on perceived needs for well-being. Results showed that COPSOQ-III dimensions of quantitative demands, recognition, sense of community, burnout, stress, and depressive symptoms, were significantly worse in our study population than the Canadian norm. Quantitative and emotional demands, health and well-being (including depressive symptoms, stress, cognitive stress, somatic stress, and burnout), and work-life conflict were also reported to have worsened since the COVID-19 restrictions for most respondents. Females and caregivers had higher odds of experiencing increased work demands, and decreased health and well-being, compared to males and non-caregivers. However, male caregivers experienced worsened supervisor relations, compared to female caregivers. Social capital also worsened for clinical and part-time employees, compared to full-time and non-clinical employees. Respondents identified increased workload support, community-building, recognition of employees' capacities and personal needs, flexible work schedules, and consistent communication, as strategies to increase well-being during COVID-19 and generally. Overall, our findings suggest that COVID-19 has increased occupational demands, work-life conflicts, and decreased well-being in veterinary academia. Institutional-level interventions are discussed and recommended to aid individual and institutional well-being.

## Introduction

Mental health is an area of concern in both the veterinary profession and in veterinary academia. Literature from the United States (US), United Kingdom (UK), and Australia report that veterinarians experience higher levels of anxiety, depression, suicidal thoughts, burnout, and lower levels of positive mental well-being, than the general population (1–5). Recent studies have contributed to a limited body of knowledge on the mental well-being of veterinarians in Canada (6) and Ontario (7). These studies yielded similar findings to their international counterparts, reporting poorer mental health and lower resilience in veterinarians than reference populations (6, 7). Comparably, mental health concerns exist in University academic institutions. Clinical and non-clinical staff, faculty, and employees in academic medical institutions also report high levels of burnout, stress, and general psychological morbidity (8–13). Studies of the mental health of veterinarians in academia are limited. However, a study of 785 Finnish veterinarians observed that those involved in education and research reported the highest levels of stress (14), and another study of 2,004 Australian veterinarians reported that those in salaried positions (i.e., research, teaching, industry and government) experienced the highest levels of depression (2).

A presumed mechanism underlying psychological morbidity in veterinarians was first proposed by Bartram et al. (1, 15), who then linked psychosocial work demands to the higher risk of suicide observed in veterinarians in the UK (5). Psychosocial work demands can be defined as interactions between work environment (i.e., social interactions), job content, organizational conditions (i.e., workplace culture), and an employee's capacity to manage these occupational factors, which can influence health, work performance, and job satisfaction (16). The occupational demands most associated with elevated stress in the Canadian veterinary population are reported to include workload and client-related issues, such as client complaints (17). Occupational stressors such as relationships with clients, euthanasia, working hours, and excessive workload, have been reported elsewhere (2, 5–7, 14, 15, 18–20).

The prevalence of work-related stressors in academia is also elevated. Increased pressure to secure external funding, diminishing resources, excessive administrative work and teaching loads, and unsatisfactory recognition and reward from administration are reported as prevalent concerns amongst non-medical academic employees (21, 22). Karasek (23) theorizes that the combination of low decision latitude (defined as an individual's control over their occupational tasks and/or work day), low social support, and high job demands is associated with greater mental and physical illnesses, including depression (24, 25), sleep disturbances (26), and cardiovascular disease (27). Given that this combination of workplace factors is observed in veterinary (1) and academic workplaces (21), gaining deeper insight into how psychosocial demands differ across clinical and non-clinical veterinarians, and research and teaching-based faculty in veterinary academia, is important to mitigate similar downstream effects.

The impact of the novel Coronavirus disease-2019 (COVID-19) pandemic on the veterinary profession is also important to consider as it has been sudden and far-reaching. Veterinarians in clinical practice have been challenged to make great modifications to help ensure patient and client safety while providing veterinary care (20). Clinical veterinary practices initially faced revenue declines associated with forced cancellation of appointments (20), followed by significant increases in work hours and weekend appointments, unpredictable schedule changes, and longer work-days to accommodate higher client-loads (28). Organizationally, economic shutdowns and new workplace policies may have accelerated infrastructure changes in academia that impact working hours, work-life balance, access to professional and personal support, and work settings, as observed with work-from-home measures (29, 30). Professionals in medical academia have also needed to adjust quickly, including canceling or restructuring research, pivoting to online teaching, and modifying clinical practice to adhere to public health guidelines while still fulfilling clinical teaching commitments and students' learning needs (31, 32). To our knowledge, no published research to date has assessed the psychosocial work demands placed on veterinary academics in both clinical and non-clinical work settings during the COVID-19 restrictions. Moreover, research on psychosocial workplace demands in the veterinarian profession has been mainly conducted outside of Canada (1, 2, 14, 19). As such, the unknown and likely widespread impacts of COVID-19 challenges in veterinary academia underscore the need for this research.

The objective of this study was to conduct a comprehensive investigation of the psychosocial work demands of employees at the Ontario Veterinary College (OVC) during COVID-19, including: workload and work pace; social relations with supervisors and colleagues; job contents including trust, recognition, and work-life conflict; and general health and well-being. Additionally, our goal was to explore the perceived needs of this population during COVID-19 to inform well-being programming at the OVC, and potentially, similar medical institutions.

## Materials and Methods

A cross-sectional study using an online questionnaire was administered *via* Qualtrics (33) from November 10, 2020, to January 10, 2021. Survey email invitations were sent to the approximately 620 staff, faculty, interns, and residents at the OVC *via* OVC listservs. A reminder email was sent ~1 month after the initial distribution of the survey. A short description of the study and a link to access the consent letter and online questionnaire was included in all electronic distributions. An announcement of the study was also posted on the OVC electronic Bulletin.

The OVC consists of four academic units (Departments of Biomedical Sciences, Clinical Studies, Pathobiology, and Population Medicine), one academic veterinary Health Sciences Centre that provides specialty care for large and small animals as well as primary care to the community, and a central administrative services department. Across these units, the OVC employs both clinical roles (i.e., veterinarians, interns, residents, faculty, registered veterinary technicians, animal care attendants, veterinary staff, agricultural assistants), and non-clinical roles (i.e., faculty, technical staff, professional staff, administrative staff). Overall, there are ~120 faculty, 200 staff in the academic and administrative departments, and 300 staff in the Health Sciences Centre.

Eligible participants were faculty members (including adjunct faculty and sessional lecturers), staff (including administrative, professional, clinical, technical, and post-doctoral fellows), and clinical residents and interns at the OVC at the time of survey. Other inclusion criteria included being 18 years of age or older and able to read and write in English. Those who had not worked during the COVID-19 pandemic (i.e., due to leave of absence, parental leave), were not eligible for inclusion. The study methodology was approved by the University of Guelph Research Ethics Board (20-08-016). Data were collected anonymously, and informed written consent was obtained before starting the questionnaire.

### Questionnaire

The questionnaire included items from the Third Version of the Copenhagen Psychosocial Questionnaire (COPSOQ-III) (34) to assess workplace psychosocial dimensions and six domains of: Demands at Work, Work Organization and Job Contents, Interpersonal Relations and Leadership, Work–Individual Interface, Social Capital, and Health and Well-being (35). Additional questions pertaining to respondent demographics, perceived changes in workplace stressors since COVID-19, and open-text questions on respondents' workplace experiences and perceived needs for well-being programming were also included.

The COPSOQ-III is an internationally validated and reliable instrument to measure psychosocial workplace factors across employee populations, workplaces, and industries (35). The COPSOQ-III facilitates a complex analysis of how work demands and psychosocial factors jointly present and impact well-being (36). The present study included 85 questions from the international middle and selected long versions of the COPSOQ-III, previously validated in the Canadian population and workforce (36). Through validated COPSOQ-III scales, 32 psychosocial dimensions were assessed across six broader psychosocial domains to assess psychosocial workplace factors, as well as health and well-being (Table 1).

**Table 1:** Definitions of the 32 dimensions within the 6 COPSOQ-III domains.

**Domain**	**Dimension**	**Definitions^a^**
Demands at work	Quantitative demands	Quantitative Demands deal with how much the employee must achieve in their work. Quantitative Demands can be assessed as an incongruity between the number of tasks and the time available to perform these tasks in a satisfactory manner.
	Work pace	Work Pace deals with the speed at which tasks must be performed and is a measure of the intensity of work.
	Emotional demands	Emotional Demands occur when the employee must deal with, or is confronted with, other people's feelings at work. “Other people” comprise both people who are not employed at the workplace (i.e., customers, clients, or students), and people employed at the workplace (i.e., colleagues, superiors, or subordinates).
	Demands for hiding emotions	Demands for Hiding Emotions occur when the employee must conceal their own feelings at work from other people. “Other people” comprise both people who are not employed at the workplace (i.e., customers, clients, or students) and people employed at the workplace (i.e., colleagues, superiors, or subordinates).
Work organization and job contents	Influence at work	Influence at Work deals with the degree to which the employee can influence aspects of work itself, ranging from, for example, planning of work to the order of tasks.
	Possibilities for development	Possibilities for Development concern whether tasks challenge the development of the employee and provide opportunities for learning. More specifically, if tasks provide opportunities for development not only in the job but also at the personal level.
	Control over working time	Control over Working Time deals with the degree of time an employee can influence conditions surrounding work (i.e., breaks, length of the working day, or work schedules).
	Meaning of work	Meaning of Work concerns both the meaning of the aim of work tasks and the meaning of the context of work tasks. The aim is “vertical,” i.e., that the work or product is related to a more general purpose, such as caring for clients or producing useful outputs. The context is “horizontal,” i.e., that one can see how ones' own work contributes to the overall product of the organization.
Interpersonal relations and leadership	Predictability	Predictability deals with the means to avoid uncertainty and insecurity. This is achieved if the employee receives the relevant information at the right time.
	Recognition	Recognition deals with recognition by the management of one's effort at work.
	Role clarity	Role Clarity deals with the employee's understanding of their role at work, i.e., content of the tasks, expectations to be met, and their responsibilities.
	Role conflicts	Role Conflicts stem from two sources. The first source is concerning possible inherent conflicting demands within a specific task. The second source is concerning possible conflicts when prioritizing different tasks.
	Illegitimate tasks	Illegitimate Tasks cover tasks that violate norms about what an employee can properly be expected to do because they are perceived as unnecessary or unreasonable; they imply a threat to one's professional identity.
	Quality of leadership	Quality of Leadership deals with the next higher managers' leadership in different contexts and domains.
	Social support from supervisor	Social Support from Supervisors deals with the employee's impressions of the possibility to obtain support from the immediate superior if they should need it.
	Social support from colleagues	Social Support from Colleagues deals with the employee's impressions of the possibility to obtain support from colleagues if they should need it.
	Sense of community at work	Sense of Community at Work concerns whether there is a feeling of belonging among employees at the workplace, i.e., if employee relations are positive, if employees work well together, and if employees feel interpersonally connected.
Work–individual interface	Job insecurity	Job Insecurity deals with aspects of employment security for the employee (i.e., regarding the risk of being fired or the certainty of being reemployed if fired).
	Insecurity over working conditions	Insecurity over Working Conditions deals with aspects of security of working conditions such as the content of work, i.e., if one is reallocated within the company, change of working hours, or deterioration of pay.
	Quality of work	Quality of Work deals with the employee's experience of the immediate output of one's work (i.e., the product made, the service accomplished/provided).
	Job satisfaction	Job Satisfaction deals with the employee's experience of satisfaction with various aspects of work.
	Work life conflict	Work Life Conflict deals with the possible consequences of work on privacy or on personal and family life and includes conflict regarding energy (mental and physical) and conflict regarding time.
Social capital	Vertical trust	Vertical Trust deals with the employee's feeling of trust in the management and vice versa. Vertical trust can be observed in the communication between the management and the employees.
	Horizontal trust	Horizontal Trust deals with the employee's feeling of trust in colleagues in daily work settings. Horizontal trust can be observed in the communication in the workplace (i.e., if one can freely express attitudes and feelings without fear of negative reactions).
	Organizational justice	Justice in the workplace refers to whether the employee feels that they are treated fairly. Four aspects are considered: first, the distribution of tasks and recognition; second, the process of sharing; third, the handling of conflicts; and fourth, the handling of suggestions from the employees.
Health and well-being	Self-rated health	Self-rated/perceived Health here concerns the employee's assessment of their own general health.
	Sleeping troubles	Sleeping Troubles here concern sleep length (determined by i.e., sleeping in, waking up and interruptions of sleep) and quality of sleep.
	Burnout	Burnout here concerns the degree of physical and mental fatigue/exhaustion of the employee.
	Stress	Stress here is defined as a reaction of the individual, and combination of tension and displeasure.
	Somatic stress	Somatic Stress here is defined as a physical health indicator of a sustained stress reaction of the individual.
	Cognitive stress	Cognitive Stress here is defined as cognitive indicators of a sustained stress reaction of the individual.
	Depressive symptoms	Depressive Symptoms cover aspects which together indicate depression.

a *Definitions as per the COPSOQ-III validation study (35)*.

For each of the COPSOQ-III dimensions, excluding self-rated health, respondents were asked whether there was a change in their answer since COVID-19 restrictions [i.e., the same since COVID-19, better now compared to before COVID-19 restrictions (i.e., the situation improved favorably), worse now compared to before COVID-19 restrictions (i.e., the situation worsened/became more unfavorable)]. For each COPSOQ-III dimension, Likert Scale–type items were measured and scaled to the interval of 0–100, as per scale instructions (35).

Demographic questions included respondent characteristics of age and gender (asked in open-text format), as well as questions on the following: current employment position at the OVC [i.e., faculty (veterinarian), faculty (non-veterinarian), registered veterinary technician (RVT), etc.]; clinical (i.e., >50% of the time is spent on clinical tasks) or non-clinical veterinary position (yes/no); employment status (full-time or part-time); weekly working hours (ranging across 10-h subscales from <24 h per week to >60 h per week); and the presence of dependents at home (yes/no; and where yes, the number of dependents per age category).

Open-text questions pertained to: the most stressful aspects of a respondent's work during the COVID-19 restrictions; what was/is helpful in the workplace during the COVID-19 restrictions; and what would help increase well-being at the OVC in general and during the COVID-19 restrictions.

### Qualitative Analysis

Common themes were identified from open-text questions based on a thematic analysis (37) of respondents' written responses. An iterative coding process was performed, as recommended for rich and reliable data (38). The six phases of thematic analysis by Braun and Clarke (37) were applied as a structured approach to developing codes and themes. In the first phase, the primary researcher (herein referred to as the “coder”) read the dataset twice for familiarization with the data. Next, the coder employed a systematic approach to construct and label codes in an inductive manner for the entirety of the data set. Here, meaningful aspects of the data and repeating patterns were identified with codes. The coder then analyzed all codes for horizontal and vertical relationships, and clustered related codes together into developing themes and subthemes, when applicable. Codes with similar or analogous content were grouped together to form the broader themes identified, and iteratively reviewed to ensure their applicability to the broader theme. To ensure reliable interpretation of the original data, the coder revised the themes and codes in relation to the entirety of the data set and coded data. Any related or overlapping themes and sub-themes were combined, and any themes, subthemes, or codes that were of low relevance to the study objectives were not retained. Through this method, key and actionable areas for well-being were identified within the entirety of the data set.

### Statistical Analyses

Descriptive statistics (i.e., means, maximums, minimums, and proportions) of demographic characteristics were summarized for all respondents.

The COPSOQ-III scales were reported as mean scores from Likert Scale–type items for each dimension. A >|9.2| difference between the mean scores of COPSOQ-III dimensions of OVC respondents and normative populations was determined as an estimate of a “minimally important difference” (39), meaning that this difference is of a magnitude that is likely to have practical consequences for workplace- and individual-level well-being.

A COPSOQ-III domain was coded as worsened if >50% of informing dimensions were reported as having worsened since COVID-19 restrictions. That is, if a participant reported that half or more of the dimensions that inform a particular domain had worsened since the COVID-19 pandemic, this domain was coded as having worsened for this participant. Alternatively, if a participant reported that more than half of the dimensions in a domain had improved or remained the same since the COVID-19 pandemic, the domain was coded as having improved/remained the same for this participant, based on suggestive data that each dimension contributes to each Domain equally (35).

Multivariable logistic regression models were separately created for each of the six psychosocial work domains of the COPSOQ-III: Demands at Work, Work Organization and Job Contents, Interpersonal Relations and Leadership, Work–Individual Interface, Social Capital, and Health and Well-being (35). Guided by previous research and a causal diagram, independent variables of interest were identified *a priori* and included: gender, hours worked during COVID-19, age, employment position (full-time vs. part-time), the presence of dependents at home, and clinical vs. non-clinical role. All independent variables were examined for collinearity using Pearson's rank correlation coefficient (40); a correlation of >|0.80| was determined as highly collinear. When two variables were highly collinear, the one with most complete data and biological plausibility was retained. Biological plausibility refers to existing biological, or in this case social models, that may explain an association, such as the number of hours worked during COVID-19 and burnout symptoms, for example (41).

To build the main effects multivariable models, univariable logistic regressions were first created to identify independent variables to be included in the main effect models with a liberal *p*-value (*p* < 0.20). Continuous independent variables were assessed for linearity with a logit transformed Locally Weighted Scatterplot Smoothing (i.e., Lowess) curve (40). If a variable was not found to be linear, it was transformed quadratically, or categorized, as needed. Each multivariable model was then built using manual backwards selection, comparing full and reduced models with Likelihood Ratio tests (40). All independent variables were assessed for confounding (defined as a 30% change in coefficients on removal of the variable) (40). If confounding was present, the variable was retained in the main effects models regardless of statistical significance. Pair-wise interactions for all the independent variables were created and tested for significance. Statistical significance was determined as *p* < 0.05. Odds ratios (OR) were reported for all independent variables maintained in the final models, with 95% confidence intervals (CIs).

Goodness-of-fit for the final models was assessed with Pearson chi-squared tests for binary data, and Hosmer-Lemeshow tests for binomial data. The influence of individual observations and covariate patterns was assessed with scatterplots of Delta-beta, Delta-deviance, and Delta-Chi-squared (40). No data were removed unless an error was clearly identified. STATA v.16 software (42) was used for all statistical analyses.

## Results

### Study Respondents

The final sample size was 157 respondents, which represents a response percentage of ~25% (157/620). No questionnaire questions were mandatory beyond the consent question, so sample sizes vary as noted. The characteristics of respondents are presented in Table 2.

**Table 2:** Demographic and career characteristics of study respondents (November 2020–January 2021).

**Characteristic**	**Total (%)**	**Mean**	**Minimum**	**Maximum**
Age (years)	154 (98%)	42	20	64
Gender	157 (100			
Female	127 (81%)			
Male	25 (16%)			
Not reported	5 (3%)			
Dependents at home	157 (100%)			
No	78 (50%)			
Yes	79 (50%)			
Up to 3 years of age	16 (10%)	1.1	1	2
4–6 years old	20 (13%)	1.2	1	2
7–12 years of age	23 (15%)	1.4	1	3
13–18 years of age	27 (17%)	1.8	1	4
Adults (18+ years of age)	20 (13%)	1.5	1	3
Current employment role				
Faculty (Veterinarian)	32 (21%)			
Faculty (Non-veterinarian)	7 (5%)			
Registered veterinary technician (RVT)	30 (19%)			
Professional staff	25 (16%)			
Administrative staff	23 (15%)			
Technical staff	7 (5%)			
Intern	6 (4%)			
Resident	4 (3%)			
Animal care assistant	5 (3%)			
Post-doctoral fellow	4 (3%)			
Doctor of veterinary science student (DVSc)	2 (1%)			
Adjunct faculty	2 (1%)			
Agricultural assistant	1 (0%)			
Sessional lecturer	1 (0%)			
Other^a^	6 (4%)			
Type of OVC position	157 (100%)			
Full-time OVC employee	143 (91%)			
Part-time OVC employee	14 (9%)			
Clinical or non-clinical position	157 (100%)			
Clinical (≥50% clinical work)	66 (42%)			
Non-clinical (<50% clinical work)	91 (58%)			
Hours worked per week (during COVID-19)	156 (99%)			
<24	10 (6%)			
25–39	76 (49%)			
40–49	30 (19%)			
50–59	19 (12%)			
≥60	21 (13%)			
Hours worked per week (prior to COVID-19)	148 (94%)			
<24	7 (5%)			
25–39	79 (53%)			
40–49	28 (18%)			
50–59	19 (13%)			
≥60	15 (10%)			

a *Other employment positions included positions related to service, research, or leadership*.

### The COPSOQ-III: Descriptive Statistics of Dimensions

The results from the COPSOQ-III are presented in Table 3 with means and 95% CIs for all dimensions, as well as normative data for international and Canadian populations for comparison (35, 43, 44).

**Table 3:** A comparison of psychosocial work demands of the OVC respondents and that of Canadian and International Workers based on COPSOQ-III dimensions.

**COPSOQ-III domain *(# of dimensions)***	**Dimension**	**Total *(n)***	**Mean dimension score of OVC study population**	**95% confidence interval *(CI)***	**Mean dimension score of Canadian workers^a^, ^d^**	**Difference in mean score of OVC and Canadian population**
Demands at work (4)	Quantitative demands	157	59.0	55.5–62.6	47.3	11.7^*^
	Work pace	157	67.4	64.3–70.6	60.4	7.0
	Emotional demands	157	54.2	50.2–58.2	50.7	3.5
	Demands for hiding emotions	157	56.5	52.7–60.3	57.0^b^	−0.5
Work organization and job contents (4)	Influence at work	157	50.3	47.0–53.6	50.0	0.3
	Possibilities for development	157	67.1	63.7–70.6	67.8	−0.7
	Control over working time	157	54.7	50.5–58.9	39^b^	15.7
	Meaning of work	157	73.4	69.7–77.1	68.5	4.9
Interpersonal relations and leadership (9)	Predictability	157	44.9	40.8–50.0	51.6	−6.7
	Recognition	156	44.4	39.4–49.3	53.7	−9.3
	Role clarity	157	72.7	69.6–75.7	67.0	5.7
	Role conflicts	157	45.9	41.7–50.2	46.2	−0.3
	Illegitimate tasks	157	44.4	39.8–49.0	45.0^b^	−0.6
	Quality of leadership	157	49.8	45.1–54.4	51.7	−1.9
	Social support from supervisor	156	58.5	54.1–62.8	63.4	−4.9
	Social support from colleagues	156	67.8	63.8–71.8	71.2	−3.4
	Sense of community at work	156	67.5	64.0–70.9	77.8	−10.3^*^
Work–individual interface (5)	Job insecurity	156	31.3	26.7–35.9	41.8	−10.5
	Insecurity over working conditions	155	22.1	18.1–26.0	19.0	3.1
	Quality of work	157	67.2	63.9–70.5	71.0^b^	−3.8
	Job satisfaction	157	64.1	61.3–66.9	69.5	−5.4
	Work life conflict	157	53.8	49.6–58.1	47.0	6.8
Social capital (3)	Vertical trust	157	59.7	55.3–64.0	61.7	−2.0
	Horizontal trust	157	65.6	61.9–69.3	62.0^b^	3.6
	Organizational justice	157	53.4	49.5–57.3	55.7	−2.3
Health and well-being (7)	Self-rated health	157	57.5	53.6–61.4	60.3	−2.8
	Sleeping troubles	157	48.3	44.5–52.1	45.1	3.2
	Burnout	157	64.9	61.2–68.6	52.5	12.4^*^
	Stress	155	56.5	53.3–59.6	45.8	10.7^*^
	Somatic stress	157	32.9	30.2–35.6	29.3	3.6
	Cognitive stress	157	43.1	39.3–46.8	35.2	7.9
	Depressive symptoms	157	41.4	37.8–45.0	21.0^c^	20.4^*^

*COPSOQ-III, Third Version of the Copenhagen Psychosocial Questionnaire; n, number of respondents.*

a *Scale means for the English core version of the COPSOQ-III from 3228 working Canadians (43).*

b *Canadian-only data not available. Scale means for the middle and long version dimensions of the COPSOQ-III from 23,361 employees in Canada, Spain, France, Germany, Sweden, and Turkey in 2016-17 (35).*

c *Canadian-only data not available. Scale mean for the selected long version dimension of the COPSOQ-II from 5,317 Danish workers in 2010 (44).*

d *Canadian and international normative data were collected prior to the COVID-19 restrictions, thus comparison with OVC data here (i.e., collected during the COVID-19 restrictions) should be interpreted accordingly.*

* *A dimension mean score >|9.2| worse for OVC employees compared to the general population (i.e., 9.2 points lower for psychosocial supports, or 9.2 points higher for psychosocial demands)i*.

Respondents responded to nearly all items, with the percentages of missing values being under 3% for all dimensions. Mean scores of psychosocial dimensions that were reported as >|9.2| worse (i.e., higher demands and/or lower supports) than the normative population, and thus potential areas of concern for workplace and personal well-being, included: quantitative demands, recognition, sense of community at work, burnout, stress, and depressive symptoms (Table 3).

### Change in COPSOQ-III Dimensions Since COVID-19

Table 4 presents respondents' perceived changes in the COPSOQ-III dimensions, including the health and well-being dimensions, since the onset of COVID-19 restrictions. The majority of respondents reported eight psychosocial dimensions as having worsened since COVID-19. These included quantitative demands, emotional demands, work life conflict, burnout, stress, somatic stress, cognitive stress, and depressive symptoms. The dimensions of burnout (% of respondents = 66%, *n* = 103), stress (69%, *n* = 108), and depressive symptoms (64%, *n* = 101) had the greatest proportion of respondents reporting that these conditions had worsened. Marginally lower proportions were reported for the dimensions of quantitative demands (% of respondents = 54%, *n* = 85), emotional demands (48%, *n* = 75), work-life conflict (48%, *n* = 75), somatic stress (54%, *n* = 85) and cognitive stress (55%, *n* = 86), as having worsened since the onset of COVID-19 restrictions. The dimension of work pace had the same proportion of respondents reporting that this dimension had either remained the same or worsened, since the onset of COVID-19 restrictions (48%, *n* = 73). The majority of respondents reported the remaining 22 psychosocial dimensions as having remained the same since the onset of COVID-19 restrictions. Role clarity (% of respondents = 87%, *n* = 136), quality of leadership (76%, *n* = 119), social support from supervisor (76%, *n* = 120), and horizontal trust (79%, *n* = 124), had the greatest proportion of respondents reporting that these dimensions remained the same since the COVID-19 restrictions. There were no psychosocial dimensions for which a majority of respondents reported an improvement since the COVID-19 restrictions.

**Table 4:** Number and proportion of respondents reporting the COPSOQ-III dimensions had improved, remained the same, or worsened, since COVID-19 restrictions.

**Domain**	**Dimension**	**Change in dimension since COVID-19 restrictions**					
		**Same since COVID-19**		**Better now compared to before COVID-19 restrictions (improved)**		**Worse now compared to before COVID-19 restrictions (worsened)**	
		***n***	**%**	***n***	**%**	***n***	**%**
Demands at work	Quantitative demands	60	38%	8	5%	85	54%
	Work pace	73	46%	8	5%	73	46%
	Emotional demands	73	46%	6	4%	75	48%
	Demands for hiding emotions	111	71%	2	1%	41	26%
Work organization and job contents	Influence at work	110	71%	17	11%	27	18%
	Possibilities for development	102	65%	7	4%	45	29%
	Control over working time	108	69%	22	14%	24	15%
	Meaning of work	116	74%	7	4%	30	19%
Interpersonal relations and leadership	Predictability	91	58%	3	2%	60	38%
	Recognition	113	72%	9	6%	32	20%
	Role clarity	136	87%	0	0%	18	11%
	Role conflicts	106	68%	4	3%	44	28%
	Illegitimate tasks	98	62%	5	3%	34	22%
	Quality of leadership	119	76%	1	1%	18	11%
	Social support from supervisor	120	76%	4	3%	30	19%
	Social support from colleagues	108	69%	4	3%	29	18%
	Sense of community at work	98	62%	7	4%	33	21%
Work–individual interface	Job insecurity	103	66%	8	5%	43	27%
	Insecurity over working conditions	94	60%	1	1%	58	37%
	Quality of work	97	62%	2	1%	33	21%
	Job satisfaction	109	69%	5	3%	40	25%
	Work life conflict	64	41%	15	10%	75	48%
Social capital	Vertical trust	110	70%	2	1%	23	15%
	Horizontal trust	124	79%	1	1%	29	18%
	Organizational justice	114	73%	0	0%	12	8%
Health and well-being	Sleeping troubles	80	51%	6	4%	68	43%
	Burnout	47	30%	4	3%	103	66%
	Stress	44	28%	2	1%	108	69%
	Somatic stress	70	45%	0	0%	85	54%
	Cognitive stress	69	44%	0	0%	86	55%
	Depressive symptoms	53	34%	1	1%	101	64%

*n, number of respondents; %, percentage of respondents from n total responders in each dimension*.

### Change in COPSOQ-III Domains Since COVID-19

Table 5 presents the proportion of respondents that reported that a COPSOQ-III domain had worsened since the onset of COVID-19 restrictions. Nearly 60% (*n* = 91) of respondents reported that Demands at Work had worsened, and nearly 70% (*n* = 109) reported worsened Health and Well-being. All other domains were reported by the majority of respondents as having remained the same since the COVID-19 restrictions.

**Table 5:** Number and proportion of respondents reporting the COPSOQ-III domains had remained the same vs. worsened, since COVID-19 restrictions.

**COPSOQ-III Domains *(# of dimensions)***	**Same compared to before COVID-19**		**Worse now compared to before COVID-19 restrictions**	
	***n***	**%**	***n***	**%**
Demands at work (4)	66	42.0	91	58.0
Work organization and job contents (4)	117	74.5	40	25.5
Interpersonal relations and leadership (9)	106	67.5	51	32.5
Work–individual interface (5)	92	58.6	65	41.4
Social capital (3)	105	66.9	52	33.1
Health and well-being (6)	48	30.6	109	69.4

### Predictors for Reporting Worsened COPSOQ-III Domain Since COVID-19

Table 6 presents the final logistic regression models assessing independent variables associated with reporting worsened COPSOQ-III domains since COVID-19 restrictions.

**Table 6:** Logistic regressions for reporting worsened COPSOQ-III domains with explanatory variables of full-time position, dependents at home, gender, clinical position, age, and hours worked per week during the COVID-19 restrictions.

**COPSOQ-III domain**	**Odds ratio**	***P*** **-value**	**95% confidence-interval *(CI)***
**Demands at work**			
Females with dependents, compared to males without dependents	13.88	0.015^*^	1.65–116.62
Males with dependents, compared to males without dependents	18.0	0.015^*^	1.75–184.68
Females without dependents, compared to males without dependents	14.76	0.013^*^	1.76–123.58
**Interpersonal relations and leadership**			
Females with dependents, compared to males with dependents	0.29	0.047^*^	0.087–0.98
Age (over 40 years old)	0.42	0.028^*^	0.19–0.91
**Work-individual interface**			
Age	0.96	0.030^*^	0.93–1.00
Full-time position	0.27	0.047^*^	0.078–0.98
Over 40 h worked during COVID-19	1.63	0.165	0.82–3.23
Dependents at home	1.44	0.339	0.68–3.06
**Social capital**			
Full-time position	0.16	0.003^*^	0.046–0.54
Clinical position	2.15	0.032^*^	1.068–4.34
**Health and well-being**			
Dependents at home	3.08	0.003^*^	1.46–6.48
Female	2.35	0.090	0.87–6.34
Over 40 h worked during COVID-19	1.66	0.202	0.76–3.60

* *Statistical significance (P-value < 0.05)*.

### Demands at Work

In the logistic regression model for the Demands at Work domain, the effects of gender and the presence of dependents at home were conditional on each other, and thus had a significant interaction. Respondents identifying as female with dependents at home had significantly greater odds of reporting worsened Demands at Work than males without dependents at home (OR: 13.88, *p* = 0.015; 95% CI: 1.65–116.62). Similarly, males with dependents at home were found to have significantly greater odds of reporting worsened Demands at Work, compared to males without dependents at home (OR: 18.0, *p* = 0.015; 95% CI: 1.75–184.68). Amongst those without dependents, female respondents had a significantly greater odds of reporting worsened Demands at Work, compared to males (OR: 14.76, *p* = 0.013; 95% CI: 1.76–123.58).

### Interpersonal Relations and Leadership

In the model for the Interpersonal Relations and Leadership domain, a similar gender-dependent interaction was observed; however, females with dependents at home had a significantly lower odds of reporting worsened Interpersonal Relations and Leadership than males with dependents at home (OR: 0.29, *p* = 0.047; 95% CI: 0.087–0.98). Age was a protective factor, with respondents older than 40 years of age being significantly less likely to report worsened Interpersonal Relations and Leadership than respondents under the age of 40 (OR: 0.42, *p* = 0.028; 95% CI: 0.19–0.91).

### Work-Individual Interface

A protective effect of age was similarly observed in the model for the Work-Individual Interface domain. Specifically, each yearly increase in age correlated to a significantly lower odds of reporting worsened Work-Individual Interface (OR: 0.96, *p* = 0.030; 95% CI: 0.93–1.00). Respondents in a full-time position were significantly less likely to report worsened Work-Individual Interface than those in part-time positions (OR: 0.27, *p* = 0.047; 95% CI: 0.078–0.98).

### Social Capital

In the model for the Social Capital domain, respondents in full-time positions had significantly lower odds of reporting that this domain worsened with COVID-19 restrictions (OR: 0.16, *p* = 0.003; 95% CI: 0.046–0.54); that is, part-time employees had increased odds of reporting worsened Social Capital. In contrast, those working in a clinical position had approximately two times greater odds of reporting significantly worsened Social Capital, compared to those in non-clinical positions (OR: 2.15, *p* = 0.032; 95% CI: 1.07–4.34).

### Health and Well-Being

Lastly, in the model for the Health and Well-being domain, respondents with dependents at home had three times greater odds of reporting significantly worsened Health and Well-being, compared to those without dependents at home (OR: 3.08, *p* = 0.003; 95% CI: 1.46–6.48). There was also a tendency for females to have a higher odds of reporting worsened Health and Well-being, compared to males, but this difference was not statistically significant (OR: 2.35, *p* = 0.090; 95%CI: 0.87–6.34).

### Qualitative Themes of Open-Text Questions

Respondents answered open-text questions regarding the most stressful aspects of their work during the COVID-19 restrictions (*n* = 110), what was helpful during the COVID-19 restrictions (*n* = 94), and what would help increase well-being at the OVC in general (*n* = 95) and during the current COVID-19 restrictions specifically (*n* = 87). Table 7 presents the themes and sub-themes derived from these data and direct exemplar quotations from respondents.

**Table 7:** Qualitative themes of respondent's open-text answers regarding workplace stressors, supports, and perceived needs to improve well-being at the OVC, during COVID-19.

**Question**	**Theme(s)**	**Sub-Themes(s)**	**Quote Examples**
What was/is the most stressful aspect(s) of your work during the COVID-19 restrictions?	(1) Childcare stressors	*1.1 Difficulty finding childcare*	“Difficulty finding childcare”
			“Resumption of normal work schedule in spring despite lack of childcare for those with children at home”
		*1.2 Role strain from balancing caregiver and work demands*	“Balancing at home childcare with working from home (how am I supposed to do these simultaneously while carrying on the same quality of work as when I was on campus?)”
			“Conflicts and time demands associated with children at home”
			“Wanting to help parent and spend time with [daughter] while also concentrating on tasks”
		*1.3 Emotional support for children*	“Emotionally supporting my children while working - especially if they have to be home due to [school/daycare] closures”
	(2) Decreased colleague social connections	“Lack of connection to colleagues, especially when trying to begin a new job”	
		“Not being with my friends at work and having the social aspect of work”	
		“Lack of face-to-face interaction with colleagues”	
		“Not connecting as often with colleagues, [or] having those hallway/doorway conversations”	
		“Not enough communication with the colleagues on the daily basis”	
	(3) Fear of exposure to COVID-19 (clinical settings)	“Worry about coming in contact with COVID, even though all precautions are in place it is still a worry as you do not know where all the people are outside of the workplace and could be bringing it into the workplace”	
		“Inability to control colleagues' private exposure”	
		“Interacting with students and residents safely - teaching in close contact but feeling unsafe and worried about catching covid”	
		“Close contact with so many students”	
		“Feeling unsafe”	
		“Worry about exposure”	
	(4) Reduced trust in colleagues	“No trust between coworkers and staff”	
		“Little support and trust from colleagues”	
	(5) Increased workload	“200–300% increased teaching load in terms of contact hours, plus everything takes longer”	
		“Everything is more difficult and takes longer”	
		“Increased case load, less people to help carry the increased case load”	
		“Increasing work demands with less staff; Impossible work demands”	
	(6) Lack of work-life balance	“Balancing work and home life”	
		“Work-life balance and separating work and life. I work at home, which is where I used to live my life”	
		“Turning off work when working from home”	
		“Living at my place of work”	
	(7) Lack of leadership support and communication	“Little to no support from supervisor/management; Unheard/dismissed concerns about difficulties in the department”	
		“Dealing with management that does not appear to listen/care”	
		“Lack of support from upper management; Unable to give feedback without fear of losing job”	
		“Not knowing, lack of information, communication and direction”	
		“Lack of communication from decision makers and lack of guidance”	
What was/is helpful in your work during the COVID-19 restrictions?	(1) Increased flexibility of work schedules	“Flexible schedules - I work a lot more weekends now and can take some time off during the week with my kids if they need”	
		“Great flexibility; Able to do different hours if needed since I'm at home”	
		“My schedule is more flexible; I don't have to be at the computer at 8:30 unless I have a meeting and I can work later”	
		“More flexibility in schedule for medical appointments”	
	(2) Social support from colleagues and supervisors	“Touch points with co-workers is valuable to socialize and also understand that challenges that many of us are facing during this time”	
		“Having good colleagues I enjoy working with”	
		“Being able to discuss issues with colleagues”	
		“Frequently virtually meeting with colleagues”	
		“The family feel of my immediate work colleagues”	
		“Having understanding colleagues”	
		“Superior support”	
		“Support from management to prioritize health”	
		“Manager's support”	
	(3) Working from home (in general)	“Being able to work from home”	
		“Comfort of pets and home environment”	
		“Being at home more with my two young children, pets, and spouse”	
		“Working from home decreases distractions of others”	
		*3.1 Lack of commute*	“Not spend time on the commute”
			“Lack of commuting time, not stressing about needing to get out the door at a certain time”
			“Less time/stress commuting”
What do you think would help increase well-being at the Ontario Veterinary College in general / during the COVID-19 pandemic?	(1) Support and advocacy for employee well-being	*1.1 Personal and mental health support*	“Personal support”
			“A general check in with people”
			“Focusing more on the people and their well-being”
			“Having a few ‘mental health’ days to use”
		*1.2 Support in clinical settings*	“More support within the clinic”
			“Less focused on clinics and filling the gaps in the schedule”
			“Front line support; Genuine interest in employee well-being”
		*1.3 Ensuring access to well-being services that don't conflict with clinical duties*	“The clinic is busy, we can't always be available to do virtual wellness, we need something in place right in the clinic”
		*1.4 Teaching & IT support*	“IT support”
			“Adjusting teaching and research to COVID-safe approaches”
		*1.5 Financial support*	“Paying residents and interns a livable wage, and giving them reasonable working hours”
			“Paying students a living wage would be an amazing first step to increasing well-being”
	(2) Recognition of employee capacities and appreciation	“More positive reinforcement and appreciation for people that work hard”	
		“Acknowledging how difficult this situation has been for all employees”	
		“Increased compassion and empathy from management”	
		“More appreciation”	
		“Genuine staff appreciation”	
	(3) Genuine engagement followed by action	“Engage with the community vs. making decisions without consultation”	
		“Allowing employees to genuinely contribute to decisions made in their specific department; Feedback and implementation of this feedback”	
		“Requesting feedback and a chance to share ideas; Regular performance/goal reviews”	
		“Actually being willing to deal with complaints and staffing issues”	
		“I think we have made great strides in talking about wellness, but I'm not sure we always walk the walk”	
	(4) Consistent and direct communication from leadership	“Increased leadership communication - updates”	
		“Consistent and clear messaging between all [Health Sciences Centre] areas”	
		“Better communication structure (too many employees hear info through the grapevine before it is formally addressed)”	
		“I find updates on the state of the college to be helpful; I like to know about the challenges we face and how we are making progress as the epidemic evolves”	
		“More and clear communication”	
		“More town hall type of updates where people are consulted and informed”	
		“Better direct communication”	
	(5) Decreased workloads	“Lighter case load”	
		“Finding a way to lighten our load”	
		“Administrative support in limiting our case load when necessary”	
		“Maintain adequate staffing levels based on case load”	
		*5.1 Fewer administrative tasks*	“More support staff - everything is downloaded to the Faculty to do”
			“Less admin work for faculty so we can go back to focusing on research and teaching”
			“Less administrative-type of work”
			“Don't increase the administrative burden of faculty, it's already more than enough”
	(6) Building a sense of community in work-related structures	“More ability to virtually socialize/build connections”	
		“Social club (i.e., movie tickets, cooking classes, plays, dinners, etc.) for employees to bring their partner/friend/family out and interact with coworkers on a personal level if they want -enhancing “community” feel)”	
		“Build a sense of community through selling our day to day wins too”	
		“We need to regain a sense of belonging”	
		“More virtual community involvement; better balance of personal virtual meetings (or non-work related meetings)”	
		“More perks and benefits to lift spirits (contests, social events, wellness activities)”	
		“Regular social events, even just coffee hours for the College, that we encourage each other to buy into/attend to show each other that it's okay to take your foot off the accelerator”	
		“Tapping into the talents of the staff and students; Building community”	
		“Music events virtually and online.music builds community”	
		“Finding ways to stay connected with each other”	

### Stressors Faced by Employees During COVID-19

The most common themes reported by respondents as work stressors during COVID-19 included: childcare (with sub-themes of difficulty finding childcare when resuming normal work schedules, role strain from balancing caregiver and work demands, and the need to emotionally support children while working); decreased opportunities to socially connect with colleagues due to working-from-home; the fear of COVID-19 exposure in clinics (i.e., physical proximity of staff, feeling unsafe when teaching residents, the inability to control other's private exposures, etc.); and reduced trust in co-workers and staff (Table 7). Increased workload (i.e., higher teaching and clinical workloads, more time required to perform similar tasks as pre-pandemic, etc.), lack of work-life balance due to work-from-home restrictions, and perceived lack of support and communication from leadership, were also themes identified as stressful aspects of respondents' work during COVID-19.

### What Was Most Helpful for Employees During COVID-19

Complementary themes were cited as being most helpful during the COVID-19 restrictions and included: the increased flexibility of work schedules and hours; the opportunity to receive social support from both colleagues and supervisors; and the ability to work from home in general. The theme of working from home in general included the sub-theme of a lack of commute to work, which respondents reported helped to decrease stress and increase free time.

### What Would Help to Increase Well-Being at Work, in General and During COVID-19 Specifically

When speaking directly to increasing well-being in general and during the COVID-19 restrictions, common themes included: more support and advocacy for employee well-being (with sub-themes of more personal and mental health support, lower workloads in clinics, greater access to well-being services that do not conflict with clinical duties, more teaching and technology support for faculty, and greater financial support for students, interns, and residents); more recognition of employee capacities and appreciation from leadership (i.e., more recognition of the high work demands caused by COVID-19, acknowledging the difficulties of COVID-19 for all employees, etc.); more genuine engagement with employees followed by action; more consistent and direct communication from leadership; and lower clinical and teaching workloads (with a sub-theme of minimizing administrative tasks for faculty). The theme of building a sense of community (i.e., through virtual socializing opportunities to build connections, creating social clubs, sharing personal stories and day-today wins of colleagues, cultivating a sense of belonging online, etc.), was also frequently reported by respondents as a positive step forward (Table 7).

## Discussion

To our knowledge, this is the first study to assess a broad range of psychosocial work demands of employees within a veterinary academic institution during the COVID-19 pandemic with validated psychometric scales. Unquestionably, demands in veterinary academia are high. We observed that the COVID-19 pandemic made worse several psychosocial demands, including home-based work challenges, increased workload and pace, reduced health and well-being, worsened interpersonal relations, and higher work demands. Below, we discuss the factors that were associated with worsened psychosocial work demands and health and well-being (including the COVID-19 pandemic, gender, caregiving, and employment characteristics), and conclude with recommendations for institutional-level interventions to aid individual- and institution-level well-being.

### COVID-19 and Work Demands

Amongst participating employees, work demands, and work pace were consistently reported as having intensified with the COVID-19 pandemic. These findings align with other recent occupational studies. For example, in May and October of 2020, two international studies with >3,000 participants in academia from 103 countries reported that the biggest change since COVID-19 was working more hours per day to accomplish the same tasks as before the pandemic (45). An increase in workload may come from the transition to digital learning, with academics reporting roughly triple the preparation time for a 1-h online lecture compared to the same lecture taught in-person (46). Increased work demands and work pace have also been reported for clinical veterinary professionals. Specifically, since the onset of COVID-19 restrictions, there has been a worldwide increase in pet ownership, increasing the demand for veterinary services such that 18% of North American practices added additional appointment slots, and 77% have needed to introduce new curbside appointments to accommodate COVID-19 social distancing policies (28). Amongst Canadian veterinary practices, 78% of have increased their client load since the onset of COVID-19, representing the greatest increase out of 18 participating countries in the international survey, including the US, UK, and Australia (28). It is expected then that 27% of participating veterinarians in the aforementioned study also reported a desire to decrease their work hours or move into a part-time veterinary position post-COVID-19 (28). A second contributing factor to the increased work demands observed here may stem from augmented employee absences, the provision of reduced or flexible work hours, or employees leaving workplaces altogether. Prior to the COVID-19 restrictions, 71% of veterinary employers in a Canadian sample said it was difficult to recruit qualified veterinary candidates, and two-thirds said they would hire additional veterinarians immediately, if candidates were available (47). As such, high workplace demands reported by veterinarians and veterinary team members were also likely affected in part by COVID-19 impacts on the availability and retention of veterinary employees.

### Female Caregivers and Work Demands

The implementation of home-based work policies with COVID-19 can particularly increase the permeability between work and family for females (48, 49). Thus, it is not surprising that we observed a gender disparity between male and female caregivers when reporting worsened work demands during the pandemic. Specifically, females with and without dependents at home had much greater odds of reporting worsened demands at work, compared to males with and without dependents. These gendered impacts are supported by international studies which report that more females than males have experienced difficulty managing their time (53 vs. 39%), balancing family, household and work responsibilities (56 vs. 44%), and managing childcare or homeschooling children (65 vs. 55%), due to COVID-19 (50). In a 2021 *Statistics Canada* report, 64% of females in the general population likewise stated that they were the parent that mostly performed homeschooling or helped children with homework during COVID-19, compared to only 19% of males, and 46% of males said that childcare duties at home were mostly their partner's responsibility (51). Importantly however, we cannot differentiate whether the 46% of males had female or male partners. Corroborated with this literature, our findings suggest that caregivers, and especially females, have experienced higher work demands due to pre-established gender roles becoming worsened by the pandemic and home-based work restrictions (52). This may have long-term career consequences. For example, female faculty members in non-medical and medical institutions (53), and especially those with children (54), were reported to be less likely to submit grant proposals and journal articles (55) or register new projects (56), since the onset of COVID-19 restrictions.

### Females, Caregivers, and Well-Being

Higher work demands, coupled with the need to balance childcare duties, may have also contributed to females and caregivers experiencing worsened health and well-being during the pandemic, as observed in the present study. Current COVID-19 research supports this, citing significantly higher levels of psychological distress in mothers of elementary school-age and younger children since the onset of the pandemic (57). Given that female representation in the veterinary and academic professions is rising in the US and Canada (58, 59), the pandemic may have increased the risk of mental health and well-being challenges for a significant portion of these professional populations. Longitudinal cohort studies assessing the long-term impacts of COVID-19-related mental health outcomes, and potential impacts on career progression and retention, would provide important insight.

### Male Caregivers and Interpersonal Relations

In contrast to our aforementioned findings of females having higher odds of negative psychosocial outcomes than males, we observed that males with dependents at home experienced significantly worsened interpersonal relations with colleagues and supervisors than females with dependents. Interpersonal support has been reported to have a more protective effect against stress and burnout for females compared to males during COVID-19 (60, 61). Herein, females may have found interprofessional supports to be more constructive when coping with childcare stressors, compared to males (61). Additionally, sense of recognition, quality of leadership, and supervisor support also inform the Interpersonal Relations and Leadership domain. While we were unable to investigate further, it may be that males faced greater adversity from supervisors, or greater personal stress to approach supervisors, when managing their new childcare roles, particularly if they had not traditionally held this role in their families. For example, requesting accommodations for childcare needs such as homeschooling, or alternative work hours to manage home duties, may have placed strain on their relations with supervisors, and downstream, their sense of recognition and support. In fact, a US study reported that although females spent more time overall on childcare, males significantly increased their childcare role during the pandemic (62), and another study in Italy reported that 40% of males spent more time on housework and 51% spent more time on childcare during COVID-19, than before pandemic restrictions (63). We presume that females faced these work-life conflicts and professional tensions prior to the COVID-19 restrictions since they already faced higher childcare duties than males pre-pandemic (57). Given that professional relations for females were not observed to decrease with the pandemic, this may indicate that females face a lower baseline of supervisor support and recognition, compared to males (64), but we do not have the data to test this hypothesis. Further research into this area would be informative.

### Clinical Professionals and Social Capital

Recent studies on Canadian veterinarians report that COVID-19 has limited job resources (i.e., co-worker or staff support, access to sufficient or high quality equipment, and readily available knowledge resources such as mentors, journals, books, etc.) (65). In the present study, lower Social Capital was reported by clinical and part-time OVC employees (of which 57% are also clinically based), compared to respondents in non-clinical and full-time roles, respectively. A lack of job resources in clinical settings may have contributed to feelings of unjust workload distribution, decreased interprofessional support, and unmet professional needs from leadership, which negatively impact the Social Capital domain. Concerns over personal health and fear of becoming infected with the COVID-19 virus were also reported as the most prevalent stressors for front-line healthcare providers caring for people during the pandemic (66). For clinical OVC employees, increased stress over teaching students and residents safely in clinics, decreased trust in colleagues, and feeling unsafe in clinical settings, were also cited as work-related stressors during COVID-19. Given that the Social Capital domain is informed by trust between colleagues and supervisors, clinical professionals may have felt increasingly concerned over whether colleagues and students were appropriately abiding by social-distancing protocols and health measures, possibly contributing to feelings of decreased interprofessional trust. With increased workplace absences since the onset of COVID-19, workload distributions may have fallen more heavily on employees continuing to work during the COVID-19 restrictions, further contributing to feelings of unfairness or distrust in the workplace. This is concerning, as social cohesion and inter-professional trust are reported as mediating factors in the prevalence of emotional exhaustion and burnout in the medical profession (67).

### Recommendations

The combination of increased workplace demands and decreased well-being observed in the present study is concerning in that it not only negatively impacts employees as individuals, but may also decrease job engagement (68), increase turnover rate and burnout (69, 70), reduce student retention (71), and challenge the ability to respond to students' needs at staff and institutional levels in academia (70). Amongst medical professionals caring for human patients, increased work demands can impact patient safety, quality of care, client satisfaction, and patient mortality rates (72, 73); it is reasonable to assume similar negative outcomes for veterinary professionals, although specific research is lacking. We recommend that academic and medical institutions focus on shaping a workplace culture that addresses occupational and psychosocial stressors to prevent similar downstream outcomes. Kumar (74) appropriately notes that too much focus on individual-level strategies does not effectively improve occupational well-being, despite them being recommended commonly by organizations. In fact, well-being interventions at the organizational-level are reported to have greater impacts on reducing burnout and promoting wellness amongst physicians, than individual-initiated interventions (75). To this end, we investigated the perceived needs of respondents for improving well-being at the OVC to help inform meaningful and institutional-level recommendations.

Respondents proposed a number of strategies to help to increase well-being during the COVID-19 restrictions and in-general (i.e., irrespective of the pandemic), including: more recognition and acknowledgment of the increased workloads faced by employees; more support for employees' personal, occupational, and financial needs; more direct communication from leadership on decisions that could help planning around COVID-19 and general College updates; more genuine employee engagement and follow-up action; and a stronger sense of community within work-related structures. Specific examples provided by respondents included more support staff to reduce workloads associated with administrative duties, providing technology assistance to improve online working conditions and support faculty with online teaching delivery, offering more mental health days, increasing wages for residents and interns, providing flexible schedules and work hours when working from home, increasing virtual social opportunities to connect with colleagues (i.e., social nights, cooking classes, music events, etc.), allowing employees to contribute to decisions made in their specific department, giving clear updates on institutional decisions that shape the domains of teaching and learning, and facilitating direct pathways for employees to voice personal needs with leadership followed by genuine recognition and action.

Systems-wide approaches such as these have been implemented in hospital-based academic centers to address similar occupational needs in medicine. Such strategies include increasing staff in administrative and well-being departments to facilitate employee recognition and manage workloads, revising institutional policies to accommodate employee support systems, increasing counseling opportunities, building inter-professional community through interdisciplinary events, and establishing frequent collaborative meetings between management and employees regarding employee needs (76). In evaluating such strategies, employees were significantly more likely to engage in wellness supports and resources, thereby contributing to employee empowerment and well-being at individual- and institutional-levels (76, 77). Difficulty accessing well-being services and scheduled times conflicting with work obligations are commonly cited barriers to employee engagement in the medical profession (78). Employees in the present study similarly indicated difficulty attending institutionally offered well-being programming due to conflicts with their clinical duties or other work responsibilities. We recommend institutions prioritize access to support services that are feasible for employees in all roles.

Often overlooked in institutional-level interventions is the importance of collaboration between leadership and employees (11), which was repeatedly indicated as desired by respondents in this study. Facilitating structural empowerment in the workplace by leadership (i.e., opportunity, information, support, resources) has been shown to contribute to improved psychological empowerment for employees (i.e., meaning, confidence, autonomy, recognition), as observed in interdisciplinary healthcare teams prior to, and during the COVID-19 pandemic (79–82). Given that aspects of structural and psychological empowerment were cited as lacking and desired by respondents here, implementing these ideals into institutional programming would be a productive starting point. Specifically, leadership training programs focused on facilitating direct communication pathways between supervisors and employees, aligning leadership and employee organizational values, empowering employees by redesigning work shifts to reflect expressed needs (i.e., flexible hours, part-time work, etc.), and developing as well as implementing targeted interventions that modify local work factors contributing to employee ill-being in organizational subunits (i.e., reducing administrative duties for faculty, decreasing clinical loads for clinicians, increasing financial support for students and residents, etc.), would be beneficial (83). In fact, focusing on specific issues raised by organizational subunits has been shown to aid in engaging and empowering employees as collaborators in shaping their occupational environment (83). More consistent communication was also reported as a common desire amongst respondents, suggesting that more frequent and consistent check-ins and updates, followed by immediate de-briefs on the concerns raised, be focused upon.

With COVID-19 disproportionately impacting females and caregivers in the present study, special consideration should also be given to increasing accessibility to policies that support caregiver needs, such as flexible work hours, sick leave, time off, and personal accommodations, as needed. Additional research funding supports (i.e., top-up funds, post-doctoral, graduate student, and/or research assistant stipends) could be made available to females and caregivers to help off-set some of the challenges they have disproportionately faced, as well as paid faculty leaves and reduced teaching loads. This said, in medical academia, the absence of clear information about eligibility, the influence of supervisor opinions, and concerns about how participation might burden coworkers or adversely impact workflow and grant funding, are cited barriers to employees engaging in work-family programs or leaves, when provided (84). Gender-neutral interventions have also been implemented by academic institutions to reduce COVID-19 impacts, such as providing tenure-track faculty with a 1-year extension, increasing research and teaching support (i.e., hiring research assistants from faculty funds, increasing support staff), establishing “COVID-19 committees” to address the impacts of COVID-19 on faculty and staff across the institution, and reducing non-essential administrative-type work (i.e., curriculum reviews, peer teaching evaluations) (85). Although such strategies may lessen work demands, gender-neutral tenure-track extensions and faculty leaves have been shown to reduce female tenure probability by 22%, while increasing male tenure probability by 19% (86), thus contributing to the already widening gender-gap caused by COVID-19. It would seem then that a cultural shift surrounding gender, caregiving, and work-family balance is needed within organizations to meaningfully implement policies and solutions that attend to female and caregiver needs.

Occupational well-being strategies would ideally also focus on fostering social cohesion and organizational resilience. For example, some medical institutions and veterinary medicine colleges offer interprofessional mentorship programs, institutionally-driven professional development opportunities, and even the ability to join faculty “teams” that engage in social events (87, 88). Formalized interventions such as these can promote community-building, increase job satisfaction, reduce occupational stress, and prevent burnout, as observed in interdisciplinary healthcare teams and academic medicine (77, 87, 89). Importantly, these strategies also help to build organizational resilience, which better equips institutions to manage adversity during times of crisis, such as COVID-19 (90). We suggest younger and part-time employees be especially involved in these programs, given that they were at greater odds of experiencing worsened sense of community during COVID-19. The current COVID-19 restrictions may present barriers to the benefits of such programs by increasing screen time and the possible risk of “Zoom fatigue” (91). However, offering regular and on-going socialization opportunities post-COVID-19 can act as a preventative measure to equip institutions with a stronger foundation of social cohesion, and in turn, organizational resilience, in the face of future workplace-altering events.

The COVID-19 pandemic did provide several benefits for employees, including increased flexibility of work schedules and hours, the ability to work from home, and decreased commuting time. Continuing to provide some flexible work conditions for employees post-COVID-19 would help to maintain these positive outcomes. This may include the development of work-life balance programs that allow broad participation and tailoring to accommodate various career tracks, as well flexible work hours and opportunities to work-from-home occasionally, if desired (84). The transition to a virtual workplace has also offered employees the opportunity to frequently connect with colleagues online, which many respondents mentioned helped to foster collegial support during COVID-19 challenges. We recommend maintaining these beneficial aspects of online workspaces to continue supporting access to rapid communication, social support, and even professional development opportunities for employees post-COVID-19. To this end, hybrid workplace models are becoming increasingly implemented into organizational structures where work is split between remote and in-office settings. This innovative change may pose potential risks to informal communication, inter-professional information seeking and collaboration (i.e., employees seeking information from online sources more than professional colleagues), and increase “Zoom fatigue” (91, 92). However, shifting from in-person to online work environments can equally improve an individual's role in a professional network. Specifically, individuals previously in peripheral positions of an in-person professional social network are more likely to occupy a more central position in an online setting (93). This suggests that hybrid models may offer improved organizational inclusion through strengthened involvement and positive workplace culture (91, 92). More research into the psychosocial outcomes of hybrid structures in academic institutions specifically would be informative.

### Limitations

This cross-sectional study assesses the psychosocial work demands and perceived needs of participating OVC employees at one moment in time. A relatively small sample size contributed to wide confidence intervals. A conservative response estimate of 25% also introduces the potential for non-response bias and under- or over-estimation of COPSOQ-III outcomes. The self-reported nature of our survey data may be subject to recall or social desirability bias when assessing pre- and post-pandemic demands. A recognized limitation of our study also arises from comparing our COPSOQ-III means to pre-COVID norms of Canadian and international working populations. Given that no COVID-19 data were available with the COPSOQ, direct comparison to other Canadian populations during the pandemic could not be made. Still, the COPSOQ-III is an internationally validated questionnaire with strong reliability and validity across diverse worker settings and workplace populations (34). Lastly, approximately one third of the sample are classified as professional or administrative staff and as such, the findings from these respondents may not be encompassing of all veterinary academia.

## Conclusion

In conclusion, this study provides novel insight into the psychosocial work demands faced by employees in a veterinary academic institution as we continue to navigate the COVID-19 pandemic. Our findings indicate that COVID-19 has increased the occupational demands and work-life conflicts, and decreased the health and well-being, of employees in our veterinary academic organization. We also noted particularly vulnerable groups, such as females, caregivers, part-time workers, and clinical employees, to be at greatest risk for experiencing worsened occupational stressors since the onset of the COVID-19 pandemic. From these findings, we suggest that evidence-based resources be implemented at the organizational-level in the OVC, and similar healthcare institutions, both during and after the COVID-19 restrictions, and offer examples. Continued research into the long-term mental health and career impacts of the COVID-19 pandemic is also critical to provide tailored well-being interventions for OVC employees, and similar professional populations. Exploring these outcomes would allow for more novel insights into how similar institutions nationally, and potentially internationally, can support clinical and academic professionals through the global transition to more online work settings.

## Data Availability Statement

The datasets presented in this article are not readily available because of ethical restrictions. Requests to access the datasets should be directed to hayleymckeee@gmail.com.

## Ethics Statement

The studies involving human participants were reviewed and approved by the University of Guelph Research Ethics Board (20-08-016). The patients/participants provided their written informed consent to participate in this study.

## Author Contributions

Material preparation and data collection were performed by HM, AJ-B, RA, BN-K, and BG. Analysis was performed by HM and BH. The first draft of the manuscript was written by HM. All authors edited subsequent versions of the manuscript, contributed to the study's conception and design, read and approved the final manuscript.

## Funding

Funding for this project was provided by the Ontario Veterinary College Dean's Office.

## Conflict of Interest

The authors declare that the research was conducted in the absence of any commercial or financial relationships that could be construed as a potential conflict of interest.

## Publisher's Note

All claims expressed in this article are solely those of the authors and do not necessarily represent those of their affiliated organizations, or those of the publisher, the editors and the reviewers. Any product that may be evaluated in this article, or claim that may be made by its manufacturer, is not guaranteed or endorsed by the publisher.
